# Deciphering the pathogenic role of rare *RAF1* heterozygous missense mutation in the late-presenting DDH

**DOI:** 10.3389/fgene.2024.1375736

**Published:** 2024-06-17

**Authors:** Yuzhao Liu, Xuesong Fan, Kun Qian, Changshun Wu, Laibo Zhang, Lin Yuan, Zhentao Man, Shuai Wu, Ping Li, Xianquan Wang, Wei Li, Yuanqing Zhang, Shui Sun, Chenxi Yu

**Affiliations:** ^1^ Department of Joint Surgery, Shandong Provincial Hospital, Cheeloo College of Medicine, Shandong University, Jinan, Shandong, China; ^2^ Shandong Mental Health Center, Shandong University, Jinan, China; ^3^ Department of Joint Surgery, Shandong Provincial Hospital Affiliated to Shandong First Medical University, Jinan, Shandong, China; ^4^ Orthopaedic Research Laboratory, Medical Science and Technology Innovation Center, Shandong First Medical University and Shandong Academy of Medical Sciences, Jinan, Shandong, China; ^5^ Digital Health Laboratory, Queen Mary Hospital, Li Ka Shing Faculty of Medicine, The University of Hong Kong, Hong Kong SAR, China

**Keywords:** DDH, RAF1 mutation, RAS-ERK pathway, validation of pathogenicity, WGS

## Abstract

**Background:**

Developmental Dysplasia of the Hip (DDH) is a skeletal disorder where late-presenting forms often escape early diagnosis, leading to limb and pain in adults. The genetic basis of DDH is not fully understood despite known genetic predispositions.

**Methods:**

We employed Whole Genome Sequencing (WGS) to explore the genetic factors in late-presenting DDH in two unrelated families, supported by phenotypic analyses and *in vitro* validation.

**Results:**

In both cases, a novel *de novo* heterozygous missense mutation in *RAF1* (c.193A>G [p.Lys65Glu]) was identified. This mutation impacted *RAF1* protein structure and function, altering downstream signaling in the Ras/ERK pathway, as demonstrated by bioinformatics, molecular dynamics simulations, and *in vitro* validations.

**Conclusion:**

This study contributes to our understanding of the genetic factors involved in DDH by identifying a novel mutation in *RAF1*. The identification of the *RAF1* mutation suggests a possible involvement of the Ras/ERK pathway in the pathogenesis of late-presenting DDH, indicating its potential role in skeletal development.

## 1 Introduction

Developmental Dysplasia of the Hip (DDH) constitutes a skeletal abnormality, characterized by a pervasive aberration in hip joint development, which is predominantly manifested through the malformation of the acetabulum or the proximal femur, ligamentous laxity, and occurrences of hip subluxation or dislocation (Kotlarsky et al., 2015). DDH has been classified into two types: early presenting and late presenting (Sharpe et al., 2006). Due to the inability to diagnose late-presenting DDH early, the incidence rate of late-presenting DDH is estimated to be approximately 1.28–2.77 per 1000 live births (Phelan et al., 2015; Pollet et al., 2017; Broadhurst et al., 2019). The optimal period for conservative treatment of DDH is within the first 6 months after birth. However, late presenting DDH often escapes diagnosis and immediate treatment postnatally, leading to prolonged complications for patients, such as chronic limping or hip pain and ultimately resulting in total hip arthroplasty for treatment (Vaquero-Picado et al., 2019). These challenges, coupled with the concealed phenotypic presentations, highlight the potential benefits of refining early diagnostic methods for late-presenting DDH. And genetic testing offers a promising approach to improve diagnostic accuracy for patients with late-presenting DDH. While late presenting DDH demonstrates genetic predisposition in its onset. However, the genetic etiology remains to be comprehensively elucidated because of its polygenic feature (Stevenson et al., 2009; Feldman et al., 2019; Harsanyi et al., 2020).

With the advancements in sequencing and experimental technologies, next-generation sequencing (NGS), including whole-exome sequencing (WES) and whole genome sequencing (WGS) has elucidated the etiologies of many congenital skeletal deformities (Goodwin et al., 2016). Xu et al., 2022 identified *KANSL1* as a novel pathogenic gene associated with DDH via WES. Similarly, Yan et al., 2022 pinpointed *LRP1* as the causative gene for DDH in a study of 68 sporadic DDH patients via WES, and they further recapitulated this human phenotype in a mouse model. Recent research unveiled numerous genetic defects associated with DDH, such as single nucleotide polymorphism (SNP) in *CX3CR1*, deletions in 18q, and deletions in 17q21 (Feldman et al., 2010; Li et al., 2017; Yu et al., 2022). While the genetic predispositions of DDH have been determined, the underlying causes for many patients remain unidentified (Kucińska-Chahwan et al., 2022).

In human embryo development, both limb skeletal and cardiac developments initiate from the lateral plate mesoderm and are modulated by analogous regulatory molecules (Prummel et al., 2020; Tani et al., 2020). Consequently, mutations in genes associated with cardiac development may have an impact on limb development. For instance, pathogenic mutations in the *TBX5* gene can lead to Holt-Oram syndrome (OMIM: #142900), while mutations in the *ZIC3* gene are known to cause VACTERL syndrome (OMIM: #142900) (Al-Qattan and Abou Al-Shaar, 2015; Reutter et al., 2016). Similarly, pathogenic mutations in the *GATA4* gene result in Tetralogy of Fallot (OMIM: #187500), and these patients may also exhibit phenotypes such as clinodactyly of the fifth finger (Abhinav et al., 2024).

The Rapidly Accelerated Fibrosarcoma 1 (RAF1), a well-studied member of the serine/threonine kinase family that includes ARAF, BRAF, and RAF1, each featuring three highly conserved functional domains—the regulatory, hinge, and kinase domain (Tran et al., 2021). RAF1 protein is primarily involved in the Ras/ERK pathway, which is closely associated with vasculogenesis and the formation of cardiac tissues (Ramos-Kuri et al., 2021; Nakhaei-Rad et al., 2023). The RAF1 protein binds to Ras protein, subsequently activating downstream proteins such as MEK1, MEK2, MAPK, ERK1, and ERK2 (Wellbrock et al., 2004; Dhillon et al., 2007). This activation is implicated in the developmental processes of the paraxial mesoderm, according to current research. The Ras/ERK pathway is a widely prevalent signaling cascade across diverse cell types, involving various growth factors such as IGF-1 and EGF, cytokines including IL-6, among others (Dorn and Force, 2005; Heineke and Molkentin, 2006).

As a key receptor in the Ras/ERK pathway, heterozygous mutations in *RAF1* are associated with the pathogenesis of Noonan syndrome (OMIM: #611553) and Leopard syndrome (OMIM: #611554), as observed in studies on autosomal dominant mutations (Pandit et al., 2007). Approximately 75% of the pathogenic *RAF1* mutations identified to date are located in the N-terminal kinase functional domain of the RAF1 protein, with 15% affecting the phosphorylation of the C-terminal residues (Tartaglia et al., 2011). Recent studies on the phenotype of RASopathies have reported joint dislocations and other skeletal deformities in patients with mutations in the Ras/ERK pathway (Tartaglia et al., 2011; Uludağ Alkaya et al., 2021). Patients with RASopathies exhibit phenotypes of limb malformations, and evidence from embryonic development suggests that *RAF1* may have a significant impact on limb development.

To elucidate the genetic etiology of late-presenting DDH, we identified a rare heterozygous missense mutation in *RAF1* in two unrelated late-presenting DDH families through WGS and Sanger sequencing. Based on the above-mentioned functions of *RAF1*, we hypothesized that *RAF1* may be the pathogenic gene of DDH. We assessed the impact of this mutation on protein structure and its interactions with various molecules using *in silico* protein structure prediction and molecular dynamics simulations. The pathogenicity of the mutation was confirmed by *in vitro* experiments that validated the effects of the missense mutation on protein expression, which were further supported by the results obtained from *in silico* analysis.

## 2 Results

### 2.1 Phenotypic analysis of unrelated DDH proband cases

**FIGURE 1:**
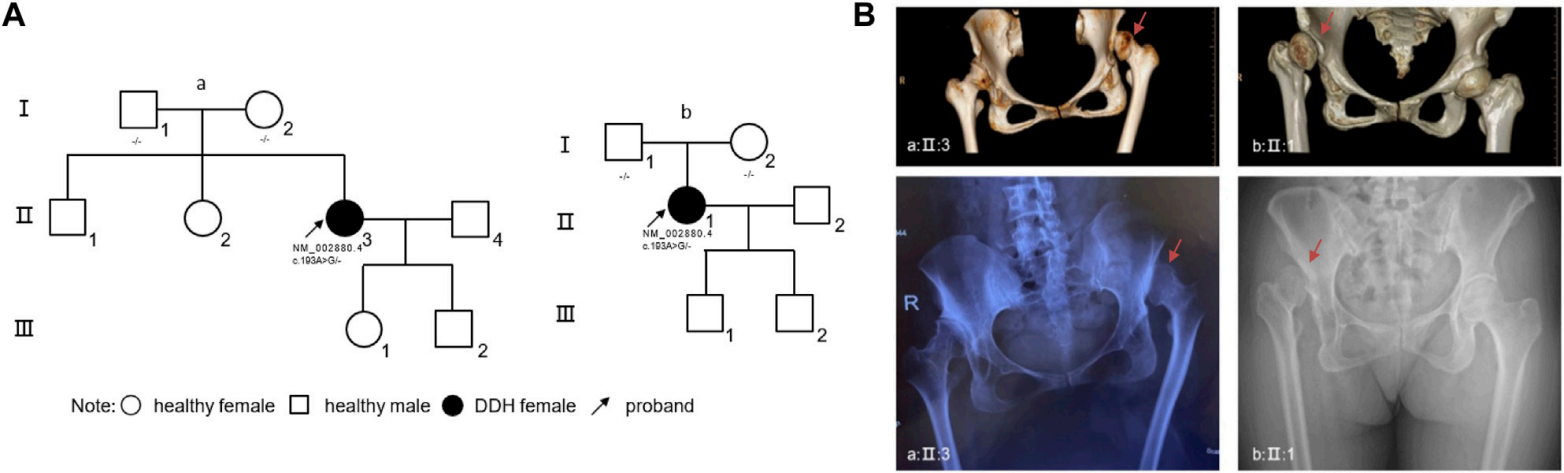
Genetic and Clinical Analysis of DDH in Unrelated Families and Radiographic Manifestations **(A)** The pedigree charts of two unrelated families (family a, associated with proband I, and family b, associated with proband II) illustrate the inheritance pattern of DDH. Circles represent females, squares represent males, filled symbols indicate individuals affected by DDH, and clear symbols represent unaffected individuals. Arrows denote the probands. Genetic mutations identified in *RAF1* are annotated beneath the corresponding individuals. **(B)** Radiographic imaging of the probands from both families. Up panels display three-dimensional computed tomography (CT) reconstructions, highlighting the dysplastic acetabula. Down panels show anteroposterior pelvic X-rays, revealing the characteristic features of DDH. Red arrow: affected hip joint. Abbreviations: R, right.

Proband I: A 46-year-old female presented with mild short stature, standing at 149 cm (1.46 standard deviations, SDs), and weighing 50 kg. The patient reported a history of limping since childhood, accompanied by unequal leg lengths. Over the past 2 years, hip pain emerged, with both pain and limping intensifying in the last 6 months. Physical examination revealed pelvic tilt, with the affected lower limb shortened by approximately 2 cm compared to the contralateral side. No webbed neck, hyperpigmentation, or facial dysmorphisms were observed upon physical examination. Electrocardiography (ECG), cardiac ultrasound, and abdominal ultrasound showed no abnormalities. Pelvic X-rays indicated bilateral hip joint dislocation, with a complete dislocation on the left side (Figure 1B). The patient and her parent denied any history of swaddling during infancy. No family history related to DDH was found in family lineage tracing (Figure 1A).

Proband II: A 49-year-old female with a height of 162 cm and weight of 57 kg. The patient noticed unequal length of lower limbs at 4 years old and experienced right hip joint pain following physical exertion 20 years ago. Examination revealed congenital dysplasia of the right hip joint. Over the past 4 years, pain has intensified following physical labor. Pelvic tilt is observed, with the affected lower limb shortened by approximately 2 cm compared to the contralateral side. While her ECG revealed sinus tachycardia, ultrasound examinations and physical assessments did not show any significant abnormalities. Pelvic X-rays displayed a total dislocation of the right hip joint (Figure 1B). Similarly, this patient also denied any history of swaddling during her early years. Also, no family history was found in this family lineage (Figure 1A).

### 2.2 Identification of heterozygous *RAF1* missense mutation

**FIGURE 2:**
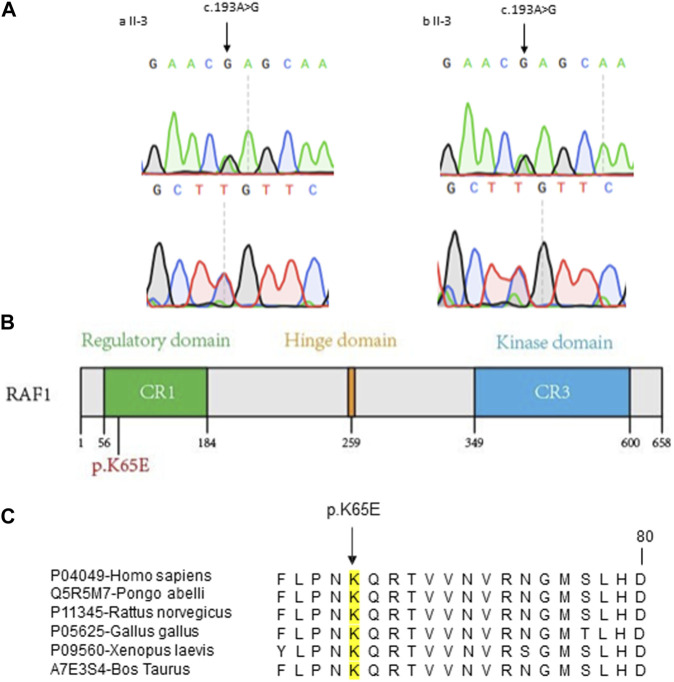
Molecular Characterization of *RAF1* Missense Mutation **(A)** Sanger sequencing chromatograms confirming the c.193A>G mutation in *RAF1* for two probands. The mutation site is marked by arrows, showing the single nucleotide substitution. **(B)** Schematic representation of the RAF1 protein domains with the location of the missense mutation c.193A>G (p.Lys65Glu) within the regulatory domain. **(C)** Cross-species conservation analysis of the RAF1 amino acid sequence surrounding the p.K65E mutation. The alignment shows the conservation of the lysine residue (K) at position 65 across various species, denoted by a black box, indicating the evolutionary conservation of this residue.

To clarify the pathogenic mutations in two patients, we initiated our investigation with Whole Genome Sequencing (WGS) of the DNA from the two patients. According to the guidelines set by the American College of Medical Genetics and Genomics (ACMG), we assessed single nucleotide variants (SNVs) and copy number variants (CNVs). Unfortunately, no pathogenic or likely pathogenic genomic variations were identified. Subsequently, we analyzed all variants of uncertain significance (VUS) and discerned that both patients harbored an identical heterozygous missense mutation (NM_002880.4: c.193A>G [p. Lys65Glu]) in the *RAF1* gene, which was confirmed by Sanger sequencing (Figure 2A). Both parents of two patients were without this mutation, the *RAF1* c.193A>G mutation was shown to be *de novo* (Supplementary Figure S1).

### 2.3 *In silico* prediction of the pathogenicity of c.193A>G mutation

To investigate the suspected pathogenicity of the detected mutation, a range of mutation prediction tools were employed to analyze its potential harm. Bioinformatic analyses revealed a substantial likelihood of pathogenicity, as reflected by the scores from several prediction instruments (SIFT = 0.06, PolyPhen = 0.816, CADD = 24.9, RawScore = 3.57). Additionally, this mutation was not observed in various population-based databases, including the 1000 Genomes Project, Exome Aggregation Consortium, and Genome Aggregation Database. Similarly, this mutation has not been reported in the Catalogue Of Somatic Mutations In Cancer database. Significantly, the mutation is situated in a region of the gene known for its conservation (Figures 2B, C).

### 2.4 Structure analysis of mutant RAF1 protein and molecular dynamic simulation

**FIGURE 3:**
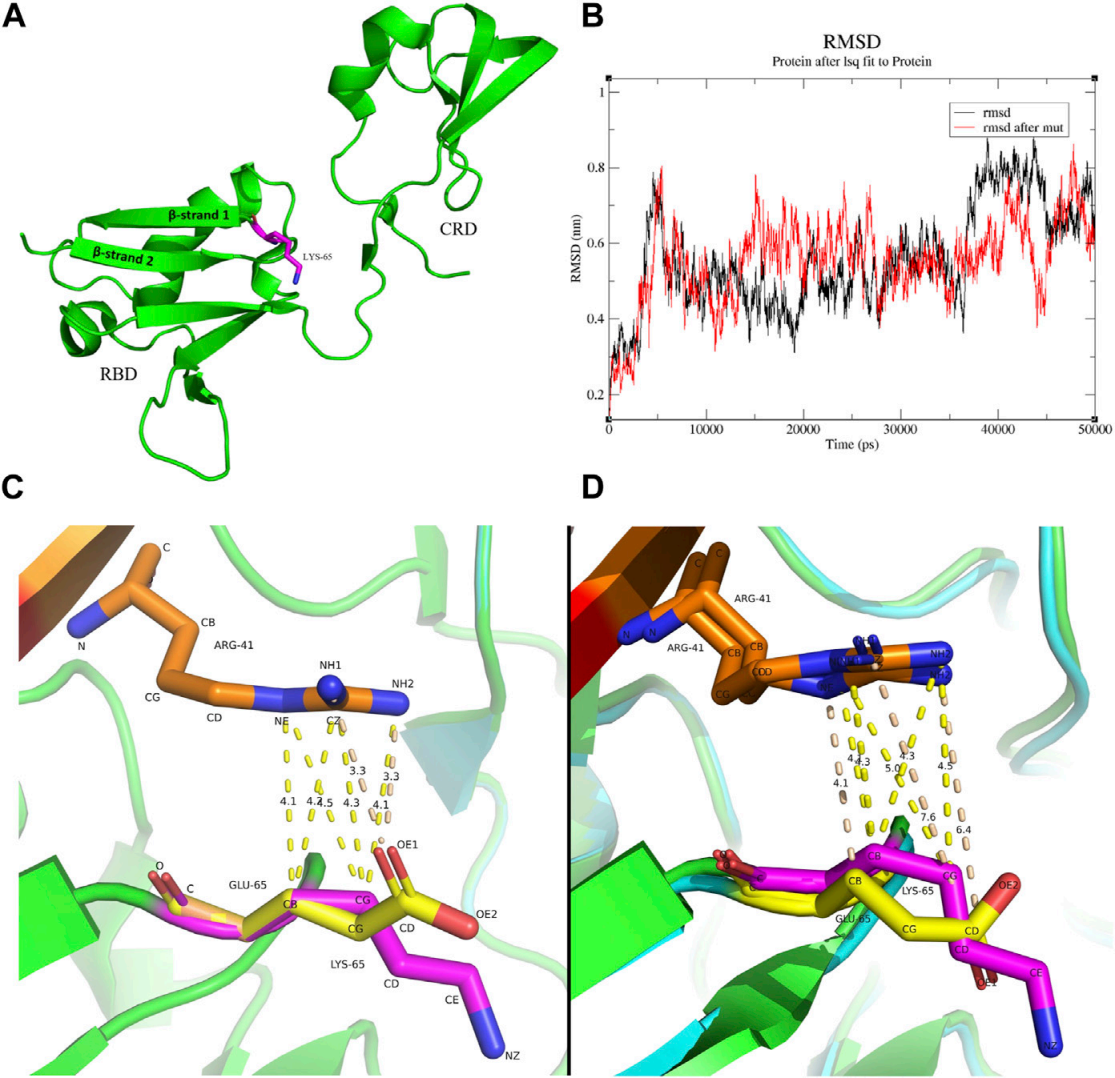
The molecular interactions between residue 41 of Kras and residue 65 of RAF1 **(A)** Position of the residues 65 in the protein structure, raw structure was got from 6XGU. **(B)** Comparison of RMSD changes in molecular dynamics simulations of the wild type and the mutant K65E, with the differences being particularly noticeable at 10 ns–20 ns and 35 ns–50 ns. **(C)** The interaction between residue 41 arginine of Kras and residue 65 of RBD before and after the mutation. Red: Kras, Orange: residue 41 arginine of Kras, Green/Cyan: RBD, Magenta: residue 65 lysine of RBD, Yellow: residue 65 glutamate of RBD. The lysine and the arginine have several contacts at distances between 4.1 Å and 4.5 Å (yellow dashed line), while the glutamate and the arginine have two contacts at a distance of 3.3 Å between O and N (wheat dashed line). **(D)** Initial protein structure of the MDS trajectory (0 ns, frame 1). Red: Kras, Orange: residue 41 arginine of Kras, Green/Cyan: RBD, Magenta: residue 65 lysine of RBD, Yellow: residue 65 glutamate of RBD. A substantial contact between the lysine and the arginine exists at a distance between 4Å and 5Å (yellow dashed line). In contrast, the glutamate and the arginine have a few contacts between 4 Å and 5 Å, while the O-N contact has changed to 7.6 Å and 6.4 Å (wheat dashed line).

To determine the impact of the mutation on the structure of the RAF1 protein and the formation of its corresponding complexes, we first simulated the structural effects of the c.193A>G mutation on the functional domain of the RAF1 protein. The mutation of lysine to glutamate at residue 65 on Ras binding domain (RBD) impedes the dynamic of RBD-cysteine-rich domain (CRD) in the absence of KRas-binding. Located at the critical junction of β-strand 1 and β-strand 2 in the RBD, residue 65 features a side-chain structure that is pivotal for the structural integrity of both the β-sheet and the linkage loop between the RBD and the CRD (Figure 3A). Comparative molecular dynamics simulations (MDS) of the wild type and the missense mutation c.193A>G (p.Lys65Glu) were conducted, with the root mean square deviation (RMSD) results indicating enhanced stability in the mutant K65E variant (Figure 3B). In the wild-type RBD-CRD structure, a tendency for the inward clustering of RBD and CRD regions was observed, leading to a more pronounced morphology of the linkage loop (Supplementary Figure S2). Conversely, the missense mutation c.193A>G (p.Lys65Glu) exhibited marked alterations in the β-sheets and loops within the RBD and CRD, but not the linkage loop (Supplementary Figure S3). It seems to indicate that, in the absence of ligand binding, the longer side-chain structure of lysine makes it more spatially flexible, while the shorter side-chain structure of glutamate suggests that it is relatively stable (Supplementary Figure S4 and Supplemental Movie).

Further, we explored the changes in the binding between mutant RAF1 and Kras proteins. The mutation of lysine to glutamate at residue 65 on RBD attenuates RBD-KRas interaction. In the wild-type configuration, C-C and C-N interactions between lysine and arginine are predominant, with distances exceeding 4 Å, while the primary interaction in the missense c.193A>G (p.Lys65Glu) is characterized by an O-N contact within a closer range of 3.5 Å (Figure 3C; Supplementary Figure S5). This indicates a transition in the RBD-Kras interaction from van der Waals forces to hydrogen bonding. However, the results of molecular dynamics simulations showed that the weak interaction force between residue 41 arginine of Kras and residue 65 lysine of RBD in the wild type persisted, whereas the hydrogen bond formed in the missense c.193A>G (p.Lys65Glu) was broken upon completion of the MDS preprocess (Figure 3D). Over the 50 ns MDS duration, the interaction forces in the wild type remained constant, while in the missense c.193A>G (p.Lys65Glu), only intermittent van der Waals forces were observed between the two residues (Supplementary Figure S6).

### 2.5 *In Vitro* validation of mutational pathogenicity

**FIGURE 4:**
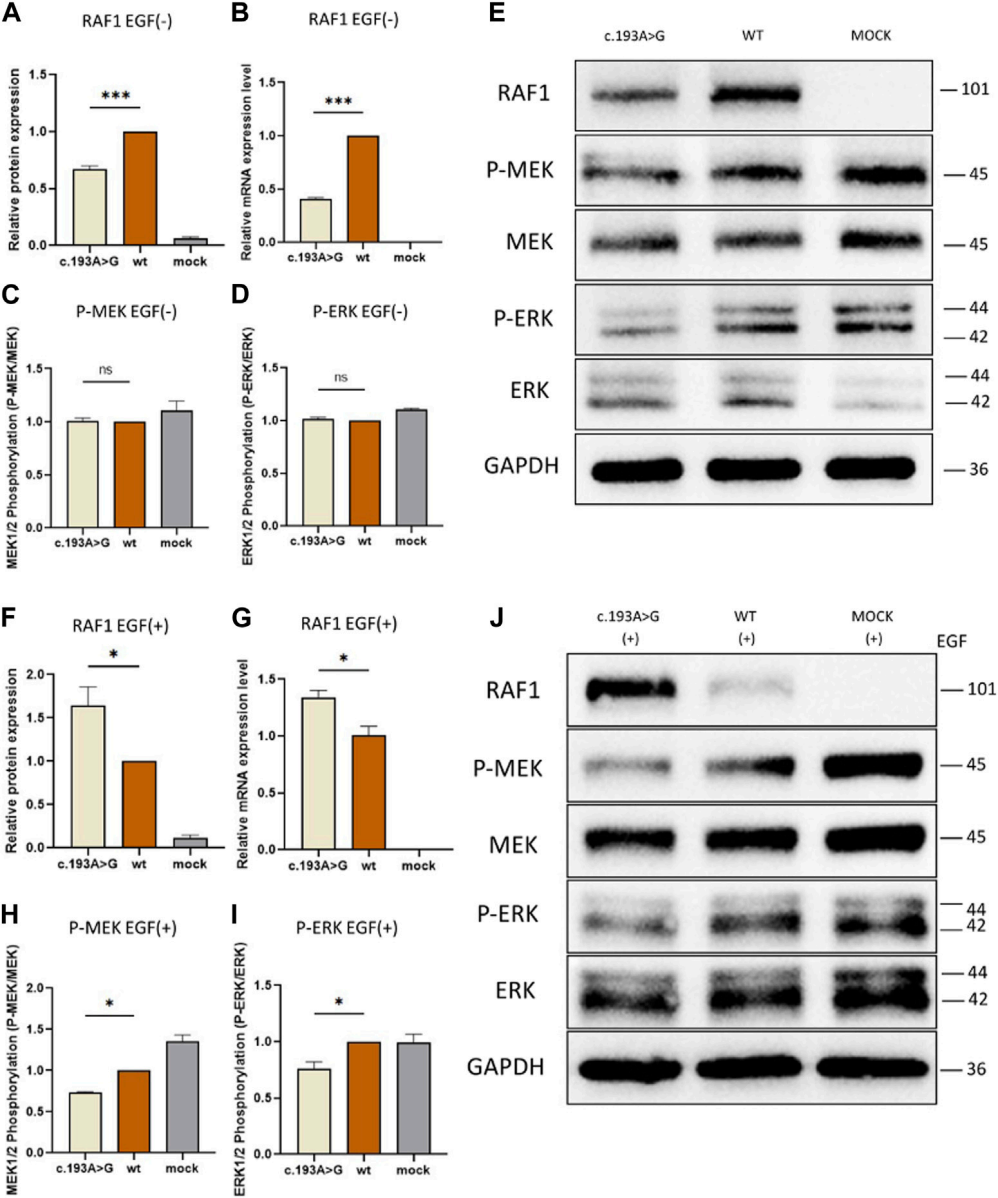
Functional Assays of RAF1 protein Expression and Downstream Signaling With and Without EGF Stimulation **(A, C, D and F, H, I)**: Quantitative Western blot analysis of downstream signaling proteins. **(A, C, D)** show RAF1 protein, phosphorylated MEK (P-MEK), and ERK (P-ERK) levels without EGF stimulation, revealing no significant difference. **(F, H, I)** depict the levels of RAF1 protein, P-MEK, and P-ERK with EGF stimulation, showing altered phosphorylation patterns in the presence of the c.193A>G mutation (*p < 0.05, **p < 0.01). **(B and G)**: Quantitative analysis of RAF1 mRNA expression levels in cells, without **(A)** and with **(F)** EGF treatment, indicating a significant decrease in mRNA levels in the presence of the c.193A>G mutation (***p < 0.001). **(E and J)**: Western blot band images representing RAF1, P-MEK, MEK, P-ERK, and ERK protein levels in cells with the c.193A>G mutation, wild-type (WT), and mock treatment. The blots include both EGF-treated (+) and untreated (−) conditions, with GAPDH serving as a loading control.

**FIGURE 5:**
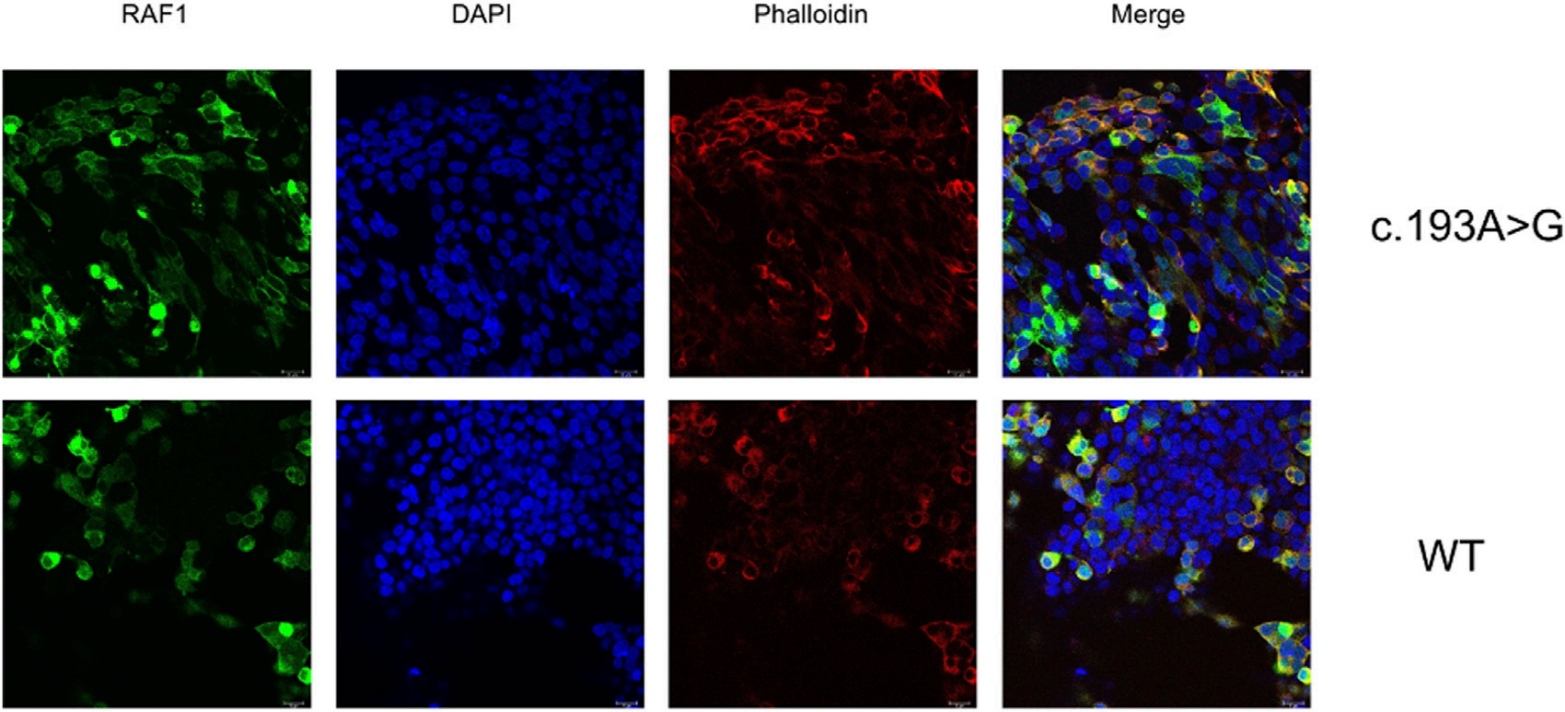
Subcellular Localization of Wild-type and Mutant RAF1 Protein in HEK 293T Cells Immunofluorescence staining demonstrates the subcellular localization of RAF1 protein harboring the c.193A>G mutation (top row) compared to wild-type (WT) cells (bottom row). RAF1 is labeled with a green fluorescent marker, nuclear DNA is stained with DAPI (blue), and F-actin is visualized using phalloidin (red). The merged images show the co-localization of RAF1 with the cytoskeletal and nuclear components. Scale bars represent 18 µm.

Our initial investigation focused on the effects of the missense mutation c.193A>G (p.Lys65Glu) on RAF1 mRNA and protein expression. In the absence of EGF stimulation, the expression levels of mutant RAF1 mRNA were significantly lower than those of the wild-type RAF1 mRNA (Figure 4B). Notably, under EGF stimulation, the expression levels of the mutant RAF1 mRNA exhibited a significant increase in comparison to the wild type (Figure 4G). Complementing this, Western blot (WB) analysis further confirmed that the missense mutation c.193A>G (p.Lys65Glu) results in enhanced RAF1 protein expression (Figure 4J). Subsequent immunofluorescence assays showed no significant differences in the intracellular localization of the mutant *versus* wild-type RAF1 proteins (Figure 5). Drawing from *in vitro* expression experiments and bioinformatic predictions, we postulate that the *RAF1* missense mutation c.193A>G (p.Lys65Glu) might contribute to disease progression by influencing RAF1 protein expression levels or by modifying its interaction with other molecules, potentially leading to dysregulation in downstream signaling pathways.

### 2.6 Effect of c.193A>G missense on RAF1 protein expression and RAF/MEK/ERK signaling pathway

To elucidate the effect of RAF1 expression alterations on downstream signaling, we examined ERK and MEK expression, as well as their phosphorylated forms, within the Ras/ERK pathway under EGF stimulation conditions. In the absence of EGF stimulation, phosphorylation levels of ERK and MEK showed no significant difference between the mutant and wild-type groups (Figures 4C–E), suggesting that the mutated RAF1 protein does not substantially influence the inactive state of the Ras/ERK pathway. Conversely, under EGF stimulation, the transducer activity of RAF1 is amplified, leading to the formation of a complex with Ras molecules and, consequently, elevated phosphorylation of downstream ERK and MEK. Notably, when compared to the wild-type RAF1 under EGF stimulation, the mutated RAF1 protein exhibits a significant increase in expression (Figures 4F, J), indirectly indicating a potential deviation from the expected complex formation with Ras. Additionally, after EGF stimulation, the phosphorylation levels of MEK and ERK in the RAF1 pathway are considerably lower in the mutant group compared to the non-mutated group (Figures 4H–J), further supporting the hypothesis that the missense mutation may impair phosphorylation of downstream molecules by altering interactions with Ras.

## 3 Discussion

*De novo* heterozygous mutations in *RAF1* (NM_002880.4:c.193A>G [p.Lys65Glu]) identified in two unrelated DDH pedigrees suggest that *RAF1* may be a novel pathogenic gene for DDH. Partial GWAS studies have linked SNPs in genes involved in cartilage differentiation, ligament formation, and heart development with DDH, such as *CX3CR1*, *ASPN*, and *TBX4* (Wang et al., 2010; Shi et al., 2011; Li et al., 2017). However, these common SNPs can only explain a small portion of the etiology in patients and are closely associated with ethnicity (Yan et al., 2022). Patients carrying heterozygous pathogenic mutations in *MYH10* present with neurodevelopmental disorders and may also have hip dysplasia (Holtz et al., 2022). Rare heterozygous mutations in *KANSL1* also exhibit an autosomal dominant phenotype of DDH (Xu et al., 2022). Heterozygous pathogenic mutations in *LRP1* also lead to early embryonic cartilage development abnormalities through the disruption of the Wnt pathway, causing autosomal dominant DDH (Yan et al., 2022). These findings suggest that the genetic pathogenic mechanisms of DDH are highly complex and likely involve multiple genes that play roles in embryonic development processes. Our finding enriches the spectrum of pathogenic genes and mutations for DDH and provides a new candidate gene implicated in this disorder.

Mutations in molecules of the Ras/ERK pathway are closely associated with a variety of skeletal phenotypes. Common skeletal deformity phenotypes in patients with Noonan syndrome include pectus deformities, cubitus valgus, and spinal malformations (Kobayashi et al., 2010; Tartaglia et al., 2011; Roberts et al., 2013; Kouz et al., 2016). Similarly, LoF mutations in the inhibitory molecules of the Ras/ERK pathway lead to non-syndromic midline craniosynostosis (Timberlake et al., 2017). Therefore, the Ras/ERK pathway might is thought to play a significant role in the development of skeletal malformations during embryonic growth, based on emerging evidence. A family-based cohort study also observed mutations in *SHC3* within the Ras/ERK pathway associated with the DDH phenotype (Dembic et al., 2023). This observation supports our findings and further substantiates the hypothesis of the critical role of the Ras/ERK pathway in embryonic skeletal development.

Pathogenic mutations located in the regulatory domain of RAF1 protein can also induce perturbation in biological function. Mutations in the RAF1 CR1 functional domain, reported in multiple large-scale heart disease cohorts, may be associated with the disease (Hoss et al., 2020; Mazzarotto et al., 2020; Hathaway et al., 2021). However, the studies related to these mutations have not conducted research on the pathogenic mechanisms (Hoss et al., 2020; Mazzarotto et al., 2020; Hathaway et al., 2021). Through molecular dynamics simulation, we discovered that the altered molecular dynamics in the mutation c.193A>G (p.Lys65Glu), evidenced by changes in β-sheets and loops within the RBD and CRD, indicate a profound impact on protein function. This alteration could disrupt downstream signaling pathways, as suggested by the changes in transducer activity and interaction forces observed in our molecular dynamics simulations. In the structure of the RAF1 protein, the amino acid at position 65 is a crucial component of the contact surface within the RBD region that interacts with RAS molecules, and mutations in amino acids of this region can significantly impact the formation of the RAS-RAF1 complex (Tran et al., 2021). Structural studies following the mutation at residue 61 of the RAF1 protein confirmed that residues 61 to 65 constitute the starting segment of the switch II region critical for the interaction between RAF1 and RBD (Fetics et al., 2015). Moreover, we observed that the c.193A>G (p.Lys65Glu) mutation leads to a tighter local binding within the Ras-RAF complex. However, molecular binding simulations revealed increased difficulties in the interaction between Ras and RAF1. These observations receive some support from *in vitro* expression experiments, although further research is needed to directly corroborate these findings. Notably, under EGF stimulation, although RAF1 expression increased, there was no significant change in the expression levels of downstream molecules in the MEK/ERK pathway.

Heterozygous LoF mutations in the *RAF1* regulatory functional domain lead to significant changes in the phosphorylation of downstream molecules in the Ras/ERK pathway. Our *in vitro* findings, including the differential expression of RAF1 mRNA and protein upon EGF stimulation, align with the hypothesis that the *RAF1* mutation influences the RAF/MEK/ERK signaling pathway. This alteration could be a crucial factor in the pathogenesis of DDH, as this pathway is integral to cell differentiation and skeletal development (Tajan et al., 2018; Motta et al., 2021). The observed changes in phosphorylation levels of ERK and MEK further support this theory. Analysis expression of RAF1 protein and phosphorylation of molecules in the pathway revealed that the mutation exhibits a LoF in the independent expression of RAF1. Without EGF stimulation, RAF1 protein expression decreases, while it increases upon EGF stimulation, but phosphorylation of downstream molecules decreases. In the Ras-ERK pathway, phosphorylation feedback regulation results in a 17-fold increase in RAF1 protein expression (Dhillon et al., 2007). This increased expression, coupled with reduced phosphorylation of downstream molecules, can be explained by the difficulty in forming the Raf1-Ras protein complex. These results allow us to confirm the pathogenicity of the mutation *in vitro*.

While current studies indicate a role for the Ras/ERK pathway in embryonic skeletal development, the specific functions within this process remain to be fully elucidated. In the process of bone maturation, hyperactivation of phosphorylation in the Ras/ERK pathway is very important for the development and differentiation of chondrocytes, leading to abnormal growth development (Tajan et al., 2018). Hence, future studies clarifying the spatiotemporal expression characteristics of the Ras/ERK pathway during embryonic development and its mechanisms in the ectoderm can better elucidate the pathogenic mechanisms of the Ras/ERK pathway.

## 4 Methods

### 4.1 Patient recruitment

Two unrelated probands and their family members were included in this study. After obtaining informed consent, clinical data and venous blood samples were collected from participating families. The collection of venous blood samples adhered to the Helsinki Declaration and was conducted in accordance with the ethics approved by the Institutional Review Boards (IRB ID: SWYXNO. 2023-013).

### 4.2 Clinical data collection and DNA extraction

Demographic information of the probands was extracted from medical records. A comprehensive and systematic physical examination was conducted on the probands, including ultrasonography of the heart and abdomen. Additionally, full-length X-rays of lower limbs and an anteroposterior view of the pelvis were taken to determine any combined skeletal deformities. The three-dimensional computed tomography (CT) reconstructions were taken to reveal dysplastic acetabula. After collecting venous blood samples from the probands and their parents, DNA was extracted following the instructions of the DNA extraction kit (TIANamp, DP348-02).

### 4.3 Whole genome sequencing and variant calling

Using the DNBSEQ-T7 (BGI, Shenzhen) sequencing platform, the DNA samples underwent 30X PCR-free whole-genome sequencing (**Supplemental Methods**). The raw data were analyzed and annotated using a previously published analytical pipeline, the Peking Union Medical College Hospital Pipeline (PUMP) (Wang et al., 2018; Chen et al., 2021; Zhao et al., 2021) (**Supplemental Methods**).

### 4.4 Variant validation and sanger sequencing

We performed an agnostic analysis of WGS data for causal variants potentially contributing to individual patients’ clinically observed phenotypes. (**Supplemental Methods**). The interpretation of causal variants was based on ACMG guidelines (Richards et al., 2015). Sanger sequencing was performed independently on available subjects and parental samples to validate variant interpretation by an orthogonal sequencing method and to investigate segregation according to Mendelian expectations for the identified variant allele(s) (Supplementary Methods, Supplementary_Table_S1).

### 4.5 Prediction of mutant protein structure and molecular dynamic simulation

Hras-RAF1 complex (PDB ID: 4G0N) and Kras-RAF1 complex (PDB ID: 6XGU) containing the changed residue were used as protein templates for before and after mutation comparisons (Fetics et al., 2015; Tran et al., 2021). All the 3-dimensional structures were visualized and preliminary analyzed using PYMOL (Version 2.5.4, Schrodinger: https://www.schrodinger.com/pymol). The UCSF Chimera (Version 1.17.3, https://www.cgl.ucsf.edu/chimera) was used for the p.Lys65Glu residue change exploration (Pettersen et al., 2004). The side chain positions were selected as the highest probability positions for none-clashes with neighboring residues obtained from the Dunbrack backbone-dependent rotamer library (Dunbrack, 2002).

GROMACS (2023.2) was used to preprocess the structure before and after mutation of the RAF protein and Kras-RAF complex and perform 50 ns MD (Abraham et al., 2015). Incomplete structures in some residues were repaired by complementary patch using PDBfixer (Version 1.9, https://github.com/pandegroup/pdbfixer) (Eastman et al., 2013). The OPLS-AA/L all-atom force field was adopted for MDS (Kaminski et al., 2001). Energy minimization, NVT equilibration, and NPT equilibration were both performed for 100 ps, respectively, using the STEEP algorithm and the leap-frog algorithm (Lemkul, 2018). All trajectories were intercepted at 20 ps intervals for visualization and analysis. FFmpeg was used to generate movies based on the intercepted trajectories.

### 4.6 Plasmid construction and cell culture

We chemically synthesized the RAF1-WT target gene fragment by adding recombination sequences before and after the target gene based on the principle of seamless cloning. Through the use of agarose gel cutting, the synthesized PCR fragments were recovered. The pEGFP-C1 plasmid vector was double digested with XhoI/BamHI, and then the RAF1-WT fragment was reconstituted into the pEGFP-C1 restriction vector. Primers were designed to amplify the RAF1-WT template (Supplemental Methods). After PCR amplification of the products using the RAF1-WT plasmid as a template, the products were recombined. After the products were transformed, the positive clones were identified and screened, and the Sanger sequencing was performed to validate the sequence of positive clones.

The HEK 293T cells (Shanghai Mcellbank Biotechnology Co., Ltd, Shanghai) were grown adherent and maintained in high-glucose Dulbecco’s modified Eagle’s medium (DMEM, Gibco, 11965092) containing 10% premium fetal bovine serum (FBS, QmSuero, mu002SR) and 1% penicillin and streptomycin (MacGene, CC004). The constructed plasmids were transfected using Lipofectamine 3000 (Invitrogen, L3000015) following the protocol suggested by the manufacturer. At 48 h after transfection, cells were starved for 24 h in the medium free of FBS and then stimulated with or without EGF (100µg/mL, Solarbio, P00033) for 15 min.

### 4.7 Detection of protein and mRNA expression

Total RNA was extracted from transiently transfected 293T cells using rapid cell RNA extraction kit (Takara Bio, 9767) and cDNA was reverse transcribed using reverse transcriptase (Takara Bio, RR047A). Quantitative PCR (qPCR) was executed using a two-step quantitative reverse transcription-PCR kit (Takara Bio, RR820A). The mRNA level was normalized to GADPH and used 2^−ΔΔCT^ method to calculate the relative mRNA level (qPCR primers were listed in Supplementary Table S2).

The protein expressions of RAF1, MEK1/2, P-MEK1/2, ERK1/2, and P-ERK1/2 were detected by western blot. Total intracellular protein was extracted using RIPA lysis buffer (Beyotime, P0013B) supplemented with 1% PMSF (Beyotime, ST507), and protein concentration was determined using the BCA Protein Concentration Assay kit (Epizyme Biotech, ZJ103). Quantified proteins were separated by sodium dodecyl sulfate-polyacrylamide gel electrophoresis followed by membrane transfer using PVDF membranes (Epizyme Biotech; WJ002). Tris-buffered saline-tween (TBST, Epizyme Biotech, PS103S) washed the PVDF membrane and blocked it with protein blocking solution for 1 hour. After washing the membranes with TBST, primary antibodies against RAF1 (1:1000, Abmart, T55225), MEK1/2 (1:5000, Abmart, T55168), P-MEK1/2 (1:1000, Abmart, TA8035), ERK1/2 (1:1000, Abmart, T40071), and P-ERK1/2 (1:1000, Abmart, TA1015) were added and overnight at 4°C. After washing PVDF membranes with TBST, horseradish peroxidase (HRP)-conjugated secondary antibodies (1:5000, Beyotime, A0216) were added and incubated for 1 hour at room temperature. The target bands were detected by an ECL chemiluminescence kit and observed in an automatic chemiluminescence image analysis system (Tanon 5200 Multi).

### 4.8 Detection of subcellular location of mutant protein

Identification of the transfected 293T cell, and detection of mutant RAF1 and wild-type RAF1 distribution in cell was performed by immunofluorescence staining. Slides were plated into six-well plates for 293T cell seeding and transfection. After 48 h, the slides were fixed with 4% paraformaldehyde (Solarbio, P1110) for 15 min and washed with PBS (Solarbio, P1020). The cells were permeabilized with Immunostaining Permeabilization Solution with Saponin (Beyotime, P0095) for 15 min and washed with PBS. After blocked with 10% immunofluorescence blocking solution for 1 hour and washed with PBS, the cells were incubated with primary antibodies against RAF1 (1:100, Abmart, T55225) at 4°C overnight. After washing with PBS, cells were incubated with CoraLite488-conjugated Goat Anti-Rabbit IgG (1:1000, Proteintech, SA00013-2) for 1 hour at room temperature. Cells were washed with PBS and stained cellular structure with phalloidin (Proteintech, PF00003), nuclei with DAPI (Beyotime, C1006). After washing with PBS, anti-fluorescence quencher was added to seal the slices. Finally, the cells were photographed using a confocal microscope (TCS SP8; Leica Microsystems, Biberach, Germany).

## Data Availability

The datasets presented in this study can be found in online repositories. The names of the repository/repositories and accession number(s) can be found in the article/Supplementary Material.
