# Supplementary material for: Deciphering the pathogenic role of rare RAF1 heterozygous missense mutation in the late-presenting DDH
Source: Front Genet. 2024 Jun 17;15:1375736. doi: 10.3389/fgene.2024.1375736 (PMC11215071; doi:10.3389/fgene.2024.1375736)
Supplement: Supplementary file 2 [file Table1.DOCX]

***Supplemental Material***

This supplemental file was intended for publication as a data supplement. The contents include the following:

[Supplemental Methods 2](#_Toc166542124)

[1. Whole Genome Sequencing 2](#_Toc166542125)

[2. Variant Calling 2](#_Toc166542126)

[3. Interpretation 2](#_Toc166542127)

[4. Primers Sequence 3](#_Toc166542128)

[Supplemental Reference 5](#_Toc166542129)

[Supplemental figures 6](#_Toc166542130)

[Supplemental_Figure_S1. Sanger Validation for Parents of Probands 6](#_Toc166542131)

[Supplemental_Figure_S2. Wild-type RBD-CRD molecular dynamics simulations 7](#_Toc166542132)

[Supplemental_Figure_S3. The mutant K65E RBD-CRD molecular dynamics simulations 8](#_Toc166542133)

[Supplemental_Figure_S4. Molecular structure of residue 65 of RBD before and after mutation. 9](#_Toc166542134)

[Supplemental_Figure_S5. Variation of intermolecular forces with distance. 10](#_Toc166542135)

[Supplemental_Figure_S6. The MDS process of contact between the residues changes in different time 11](#_Toc166542136)

[Supplemental Tables 12](#_Toc166542137)

[Table S1 PCR reaction liquid composition（50μL）and Template setting. 12](#_Toc166542138)

# Supplemental Methods

## Whole Genome Sequencing

WGS was performed according to following steps: the quality-controlled genomic DNA was randomly fragmented. The fragmented genomic DNA was selected to a certain average size. The fragments were subjected to end-repair and then was 3’ adenylated. And then adaptors were ligated to the ends of these 3’ adenylated fragments. The corresponding library quality control protocol will be selected depending upon product requirements. Single-stranded adaptor ligation products are produced via denaturation. The reaction system and program for circularization are subsequently configured and set up. Single-stranded cyclized products are produced, while uncyclized linear DNA molecules are digested. Single-stranded circle DNA molecules are replicated via rolling cycle amplification, and a DNA nanoball (DNB) which contains multiple copies of DNA is generated. Sufficient quality DNBs are then loaded into patterned nanoarrays using a high-intensity DNA nanochip technique and sequenced through combinatorial Probe-Anchor Synthesis.

## Variant Calling

The sequencing data were analyzed and annotated using an in-house developed analytical pipeline, Peking Union Medical College hospital Pipeline (PUMP) ^1,2^. Paired sequences obtained from each sample were aligned to the GRCh37/hg19 human reference sequence using Burrows-Wheeler Aligner (BWA) with the MEM algorithm. BAM files were generated by Picard. Sequence reads were recalibrated by Realigner Target Creator in Genome Analysis Toolkit (GATK) and sequence variants were called by GATK Haplotype Caller. Annotation of de novo, compound heterozygous, and recessive inherited variants were calculated with Gemini (version 0.19.1) for in silico subtraction of parental variants from the proband’s variants, with accounting for read number information extracted from BAM files. Computational prediction tools (GERP++ ^3^, Combined Annotation Dependent Depletion (CADD) ^4^, SIFT ^5^, and Polyphen-2 ^6^) were used to predict the conservation and pathogenicity of candidate variants. Population frequency of each variant was obtained from publicly available databases such as the 1000 Genomes Project (http://www.internationalgenome.org/), the Exome variant server, NHLBI GO Exome Sequencing Project (ESP) (http://evs.gs.washington.edu/EVS/), the Exome Aggregation Consortium (ExAC) (http://exac.broadinstitute.org/), and genome Aggregation Database (gnomAD , http://gnomad.broadinstitute.org/).

## Interpretation

All variants were first filtered against a general population frequency of 0.01 based on 1000 Genomes, ExAC, and gnomAD databases. Intronic/UTR variants outside canonical splicing sites and synonymous variants without functional reports.

Evaluation of the pathogenicity of the variants was based on the American College of Medical Genetics and Genomics (ACMG) guidelines ^7^. All pathogenic and likely pathogenic variants were manually reviewed using Integrative Genomics Viewer (IGV). The following categories of supportive evidence were collected for each susceptive variant:

- Very strong evidence 1 (PVS1): Protein-truncating variants (nonsense, frameshift, canonical splice sites, or start codon) in genes where loss-of-function (LoF) is a known pathogenic mechanism of the disease.
- Strong evidence 1 (PS1): Variants which result in the same amino acid changes with established pathogenic variants.
- Strong evidence 2 (PS2): Variants which arise *de novo* in a trio family where both parents are unaffected.
- Strong evidence 3 (PS3): Variants with well-designed *in vitro* or *in vivo* experiment to validate their function.
- Moderate evidence 1 (PM1): Variants located in mutational hot spot or well-validated functional domain of the proteins.
- Moderate evidence 2 (PM2): Absent or at extremely low frequency (for recessive genes) from population database (gnomAD, ExAC)
- Moderate evidence 3 (PM3): Variants with another pathogenic/likely pathogenic variant *in trans* for recessive genes.
- Moderate evidence 4 (PM4): Variants which result in changes in protein length, such as in-frame indels and stop-loss variants.
- Supporting evidence 1 (PP1): Variants co-segregated with disease in families with multiple affected members.
- Supporting evidence 2 (PP2): Missense variants in a gene in which missense variants are highly suspected to be deleterious.
- Supporting evidence 3 (PP3): Variants predicted to be deleterious by multiple *in silico* prediction tools.
- Supporting evidence 4 (PP4): Phenotypes of the patient is highly specific to the manifestation of the disease.

Then, variant pathogenicity was decided according to the existence and combination of the supportive evidence:

- Pathogenic variant: a) one very strong evidence AND one strong evidence; b) one very strong evidence AND two moderate evidence; c) one strong evidence AND three moderate evidence.
- Likely pathogenic variant: a) one very strong evidence AND one moderate evidence; b) one strong evidence AND one moderate evidence; c) three moderate evidence; d) two moderate evidence AND two supporting evidence.

After the selection of pathogenic or likely pathogenic alleles, anticipated mode of inheritance associated with the identified genes was then considered. For dominant or X-linked dominant genes, a heterozygous (or hemizygous) variant is sufficient to be potentially disease-causing. For genes usually associated with an autosomal recessive disease trait inheritance, biallelic variants revealed through trio exome sequencing were required to suspect a gene to be disease-causing.

If the observed variant(s) is/are pathogenic and consistent with the expected mode of inheritance, gene-related phenotypes were compared to the patient phenotype. A positive molecular finding is defined when the phenotypic spectrum of the gene could explain the whole clinical presentation of the patient.

## Primers Sequence

For plasmid construction:

Forward: TGCCGAACGAGCAAAGAACAGTGGTCAATGTGC

Reverse: TCTTTGCTCGTTCGGCAAGAAAACACGGATAG

For RAF1 PCR:

Forward: AATGTGCTCCACAGGCAGAT
Reverse: GATGCCGTGTTTGATGGCTC

For RAF1 qPCR:

Forward: GGGAGCTTGGAAGACGATCAG
Reverse: ACACGGATAGTGTTGCTTGTC

For GAPDH qPCR:

Forward: CCCAGACAGTGGATGATGC

Reverse: TTGTCCTTCCTCCCAGGTC

# Supplemental Reference

1. Zhao S, Zhang Y, Chen W, et al. Diagnostic yield and clinical impact of exome sequencing in early-onset scoliosis (EOS). *J Med Genet* 2021; **58**(1): 41-7.

2. Wang K, Zhao S, Liu B, et al. Perturbations of BMP/TGF-beta and VEGF/VEGFR signalling pathways in non-syndromic sporadic brain arteriovenous malformations (BAVM). *J Med Genet* 2018; **55**(10): 675-84.

3. Davydov EV, Goode DL, Sirota M, Cooper GM, Sidow A, Batzoglou S. Identifying a high fraction of the human genome to be under selective constraint using GERP++. *PLoS computational biology* 2010; **6**(12): e1001025.

4. Kircher M, Witten DM, Jain P, O'Roak BJ, Cooper GM, Shendure J. A general framework for estimating the relative pathogenicity of human genetic variants. *Nat Genet* 2014; **46**(3): 310-5.

5. Vaser R, Adusumalli S, Leng SN, Sikic M, Ng PC. SIFT missense predictions for genomes. *Nature protocols* 2016; **11**(1): 1-9.

6. Adzhubei IA, Schmidt S, Peshkin L, et al. A method and server for predicting damaging missense mutations. *Nature methods* 2010; **7**(4): 248-9.

7. Richards S, Aziz N, Bale S, et al. Standards and guidelines for the interpretation of sequence variants: a joint consensus recommendation of the American College of Medical Genetics and Genomics and the Association for Molecular Pathology. *Genet Med* 2015; **17**(5): 405-24.

# Supplemental figures

## Supplemental_Figure_S1. Sanger Validation for Parents of Probands


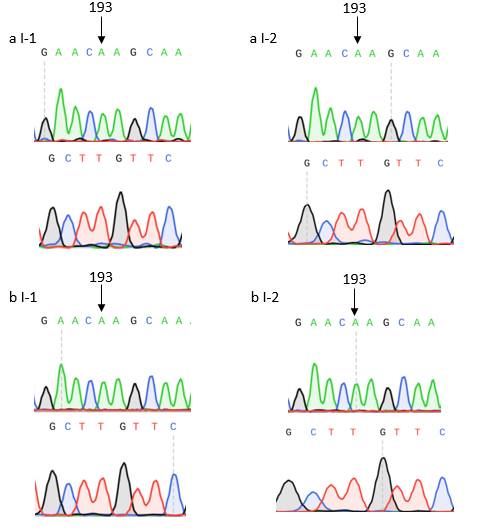


Sanger sequencing validation results for parents (a-I1, a-I2, b-I1, b-I2) of 2 unrelated probands.

## Supplemental_Figure_S2. Wild-type RBD-CRD molecular dynamics simulations


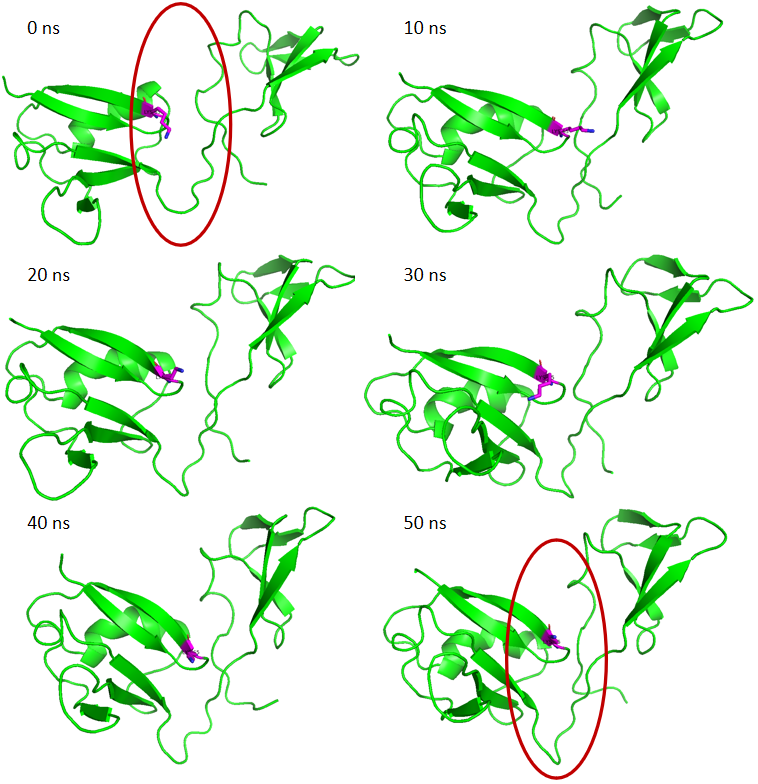


Wild-type RBD-CRD molecular dynamics simulations are shown as 10 ns interval intercepts, with red circles marking the linkage loop changes before and after the simulation.

## Supplemental_Figure_S3. The mutant K65E RBD-CRD molecular dynamics simulations


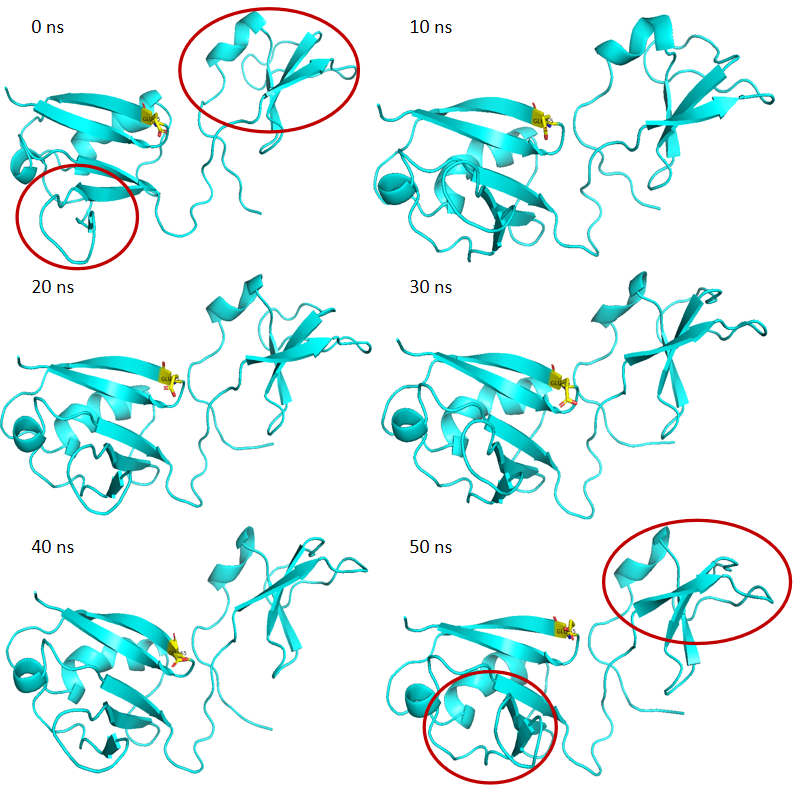


The mutant K65E RBD-CRD molecular dynamics simulations are shown as intercepts at 10 ns intervals, with red circles marking the changes in the RBD rigion and CRD region before and after the simulation.

## Supplemental_Figure_S4. Molecular structure of residue 65 of RBD before and after mutation.


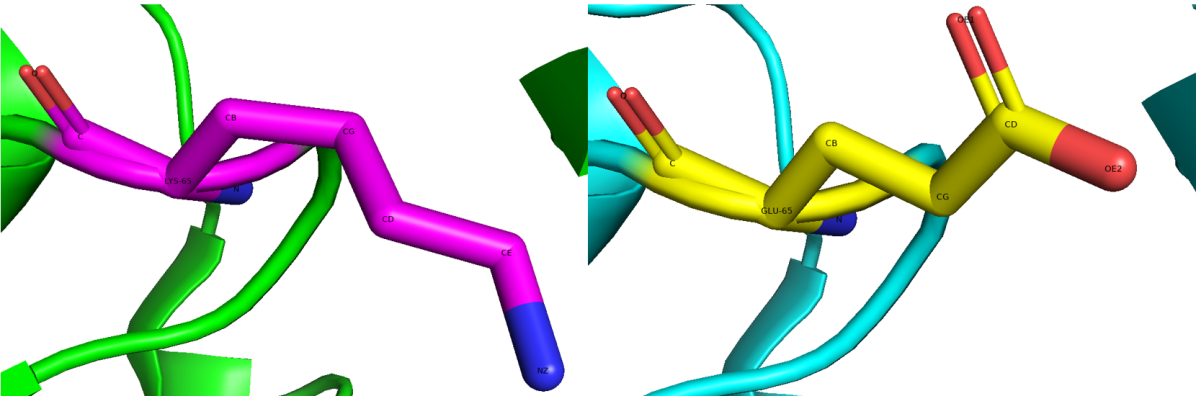


The left panel displays the original RAF1 structure. The right panel illustrates the mutated RAF1 structure, highlighting the position change of the amino acid residue GLU65. The mutation induces a notable conformational shift affecting the spatial arrangement of the surrounding residues.

## Supplemental_Figure_S5. Variation of intermolecular forces with distance.


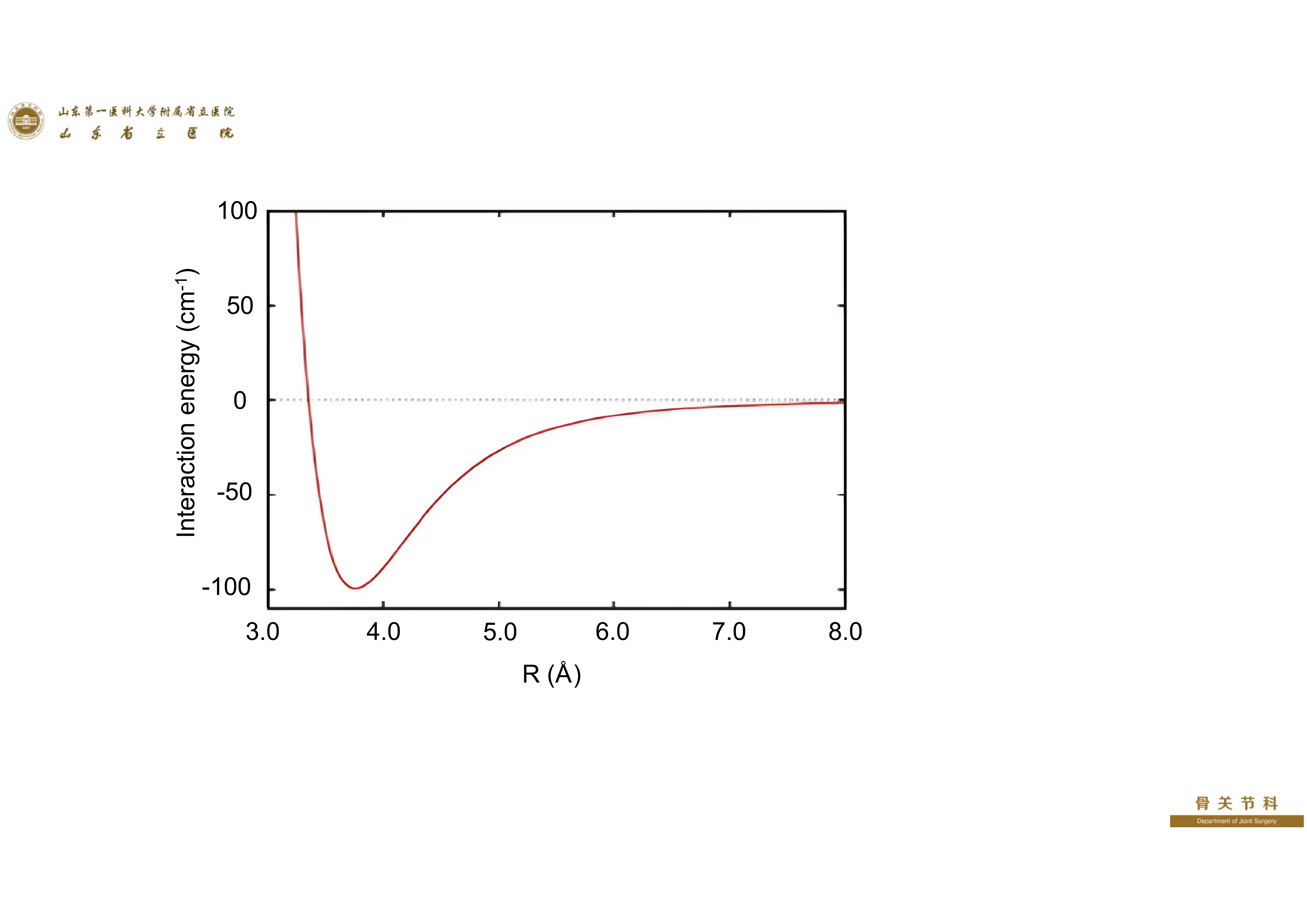


The graph shows the interaction energy as a function of distance (R) in angstroms (Å) The red curve represents the van der Waals attraction. When the molecules approach each other, the van der Waals attraction decreases the energy of the system, most significantly at distances of 3.5Å to 4Å; as the molecules approach further, the repulsive force increases rapidly, increasing the energy of the system.

## Supplemental_Figure_S6. The MDS process of contact between the residues changes in different time


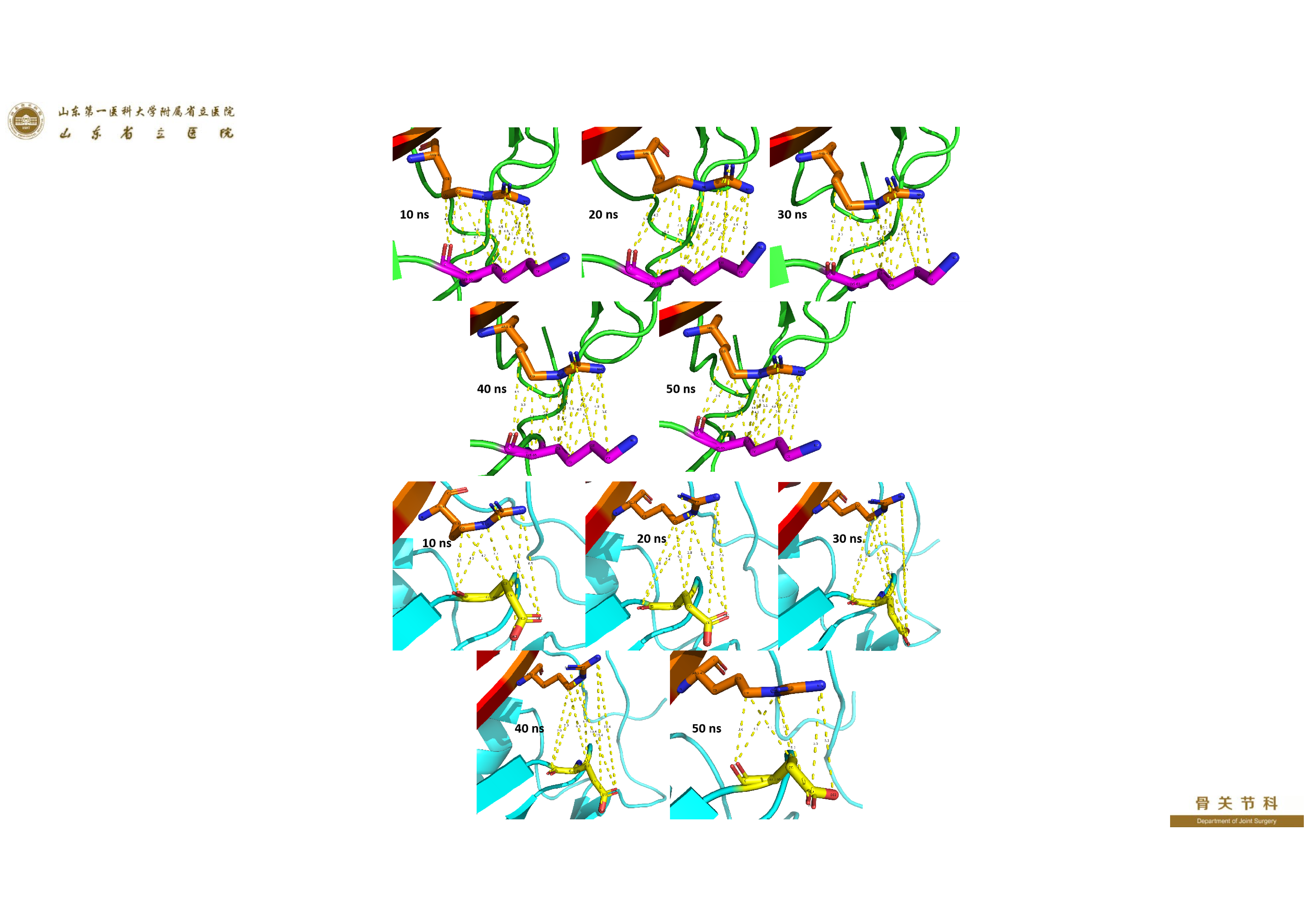


MDS process of contact between the residues varies over the course of 10 ns, 20 ns, 30 ns, 40 ns, and 50 ns, in that order. Oringe: residue 41 arginine of KRas, Green/Cyan: RBD, Magenta: residue 65 lysine of RBD, Yellow: residue 65 glutamate of RBD.

# Supplemental Tables

## Table S1 PCR reaction liquid composition（50μL）and Template setting.

| Template DNA | Primer1（10μM） | Primer2（10μM） | dNTPs（2.5 mM） | 10×EX Taq BUffer （20 mM） | TAKaRa Ex Taq（5U/μL） | D/RNase-Free Water |
| --- | --- | --- | --- | --- | --- | --- |
| 200ng | 1μL | 1μL | 4μL | 5μL | 0.25μL | Up to 50μL |

| predegeneration | circulation | | | Terminal extension | save |
| --- | --- | --- | --- | --- | --- |
|  | degeneration | annealing | extension |  |  |
| 98°C | 98­°C | 56­°C | 72­°C | 72­°C | 16­°C |
| 2min | 10s | 30s | 1min | 7min |  |
| 1 cycle | 30 cycle | | | 1 cycle | 1 cycle |
